# The comparison of visual outcomes, aberrations, and Bowman’s layer micro-distortions after femtosecond laser small-incision lenticule extraction (SMILE) for the correction of high and moderate myopia and myopic astigmatism

**DOI:** 10.1186/s12886-019-1135-9

**Published:** 2019-06-27

**Authors:** Bing Qin, Jing Zhao, Meiyan Li, Peijun Yao, Xingtao Zhou

**Affiliations:** Department of Ophthalmology and Optometry, Eye and ENT Hospital, Fudan University, Shanghai, China Shanghai Research Center of Ophthalmology and Optometry, NHC Key Laboratory of Myopia, Shanghai, People’s Republic of China

**Keywords:** Femtosecond laser small-incision lenticule extraction, Higher-order aberration, Micro-distortion

## Abstract

**Background:**

This study compares the clinical outcomes of femtosecond laser small-incision lenticule extraction (SMILE) for the correction of myopia and myopic astigmatism greater than − 10 D, and − 10 D or less respectively.

**Methods:**

60 eyes/patients were equally selected into group 1 (myopia and myopic astigmatism of − 10 D or less) and group 2 (myopia and myopic astigmatism of over − 10 D), both of which were treated with SMILE. Visual and refractive outcomes, corneal higher-order aberrations, and Bowman’s layer micro-distortions were evaluated preoperatively, 3 months, and 6 months postoperatively.

**Results:**

LogMAR corrected distance visual acuity (CDVA) of group 1 and group 2 was − 0.069 ± 0.047 and − 0.053 ± 0.073 6 months postoperatively (*P* = 0.48). 100% eyes in group 1 and 97% in group 2 were within 1 D of targeted correction (*P* = 0.45). Meanwhile, 100% eyes in group 1 and 97% in group 2 had an uncorrected distance visual acuity of 20/25 or better (*P* = 0.20). Changes in corneal higher-order aberrations root mean square, coma, and trefoil were similar between the two groups but spherical aberration was higher in group 2 (*P* < 0.01). Micro-distortions were observed in 53% in group 1 and 77% in group 2. More micro-distortions were observed in group 2 (3.40 ± 2.66) than in group 1 (2.07 ± 2.29) (*P* = 0.041). The total number of micro-distortions was not correlated with postoperative CDVA (*P* = 0.77).

**Conclusions:**

Visual outcomes showed similar results of SMILE for myopic correction of > − 10 D and ≤ − 10 D. Refractive outcomes showed slightly under-correction in higher myopic eyes. Higher myopic treatment tends to induce more spherical aberrations. Micro-distortions had no impact in visual and refractive outcomes.

## Background

Femtosecond laser small-incision lenticule extraction (SMILE) is a newly developed refractive surgical technique, which utilizes femtosecond laser to cut no corneal flap but a stromal lenticule and extracted through a small side cut [1–3]. Many existing studies have found that compared with laser in situ keratomileusis (LASIK), SMILE have similar surgical outcomes in terms of safety, efficacy, predictability, and corneal higher-order aberrations [4–6].

Standard SMILE procedure is capable of correcting myopia up to − 10 diopters (D) using Visumax® femtosecond laser system. With the recent software upgrade, it also made myopic correction of over − 10 D possible. The aim of this study was to compare the visual and refractive outcomes, higher-order aberrations, and corneal morphological change of SMILE for the treatment of myopia and myopic astigmatism of > − 10 D with those ≤ − 10 D performed by the same experienced surgeon.

## Methods

Sixty patients were consecutively enrolled in this prospective study. All patients underwent routine preoperative examinations and met the surgical indications for SMILE. The SMILE procedures were conducted in the refractive surgery center of the department of ophthalmology, Eye and ENT Hospital of Fudan University (Shanghai, People’s Republic of China) from October 2015 to February 2016. Inclusion criteria are: ages between 18 to 40 years old, a corrected distance visual acuity (CDVA) of 20/25 or better (≥0.1 logMAR), a stable refractive error (≤0.50 D of refractive error change in the past 2 years), and no contact lens use for 2 weeks prior preoperative examinations. Exclusion criteria include keratoconus or suspicion of keratoconus, severe dry eye, corneal scar, and a history of herpetic keratitis, cataract, glaucoma, retinal pathology, ocular surgery, or any systemic disease. Eyes with a calculated postoperative residual stromal bed thickness of less than 250 μm were also excluded.

A random eye of each patient was selected for data analyses. Patients were divided into group 1 and group 2 according to preoperative manifest refraction. In group 1 (30 eyes of 30 patients), all eyes have the sum of spherical refraction and astigmatism of − 10 D or less. In group 2 (30 eyes of 30 patients), all eyes have the sum of spherical refraction and astigmatism of over − 10 D, as well as the spherical refraction of − 7.5 D or more. Demographic, preoperative and surgical information were summarized in Table 1. This study adhered to the tenets of the Declaration of Helsinki and was approved by the Ethics Committee of the Eye and ENT Hospital, Fudan University. All patients signed their informed consent after a detailed explanation of the risks and potential outcomes of refractive surgery and the study.

**Table 1:** Demographic, preoperative and surgical information

Mean ± SD (range)	group 1	group 2	*P* Value
Sex (female/male)	18/12	22/8	0.31
Age (yrs)	24.50 ± 6.81 (18~45)	26.87 ± 6.09 (19~38)	0.31
Manifest spherical equivalent (D)	−6.40 ± 1.29 (−8.25~ − 3.50)	− 10.06 ± 0.77 (− 11.75~ − 8.63)	< 0.01
Manifest cylinder (D)	−0.79 ± 0.60 (− 3.00~0)	−1.24 ± 1.05 (− 4.50~0)	0.045
Preoperative LogMAR CDVA	0.023 ± 0.049 (− 0.1~0.1)	0.00 ± 0.045 (− 0.1~0.1)	0.091
Preoperative CCT (μm)	540.54 ± 30.08 (498~599)	547.17 ± 25.09 (503~599)	0.34
Lenticule thickness (μm)	126.69 ± 21.24 (75~136)	151.67 ± 4.79 (140~158)	< 0.01
Residual stromal thickness (μm)	293.86 ± 35.60 (277~369)	280.83 ± 18.73 (257~322)	0.065
Optical zone (mm)	6.48 ± 0.032 (6.0~6.6)	6.12 ± 0.14 (6.0~6.5)	< 0.01

*SD* = Standard deviation; *D* = Diopters; group 1 = small-incision lenticule extraction for myopia and myopic astigmatism *≤10 D; group 2* = small-incision lenticule extraction for myopia and myopic astigmatism > *− 10 D; LogMAR* = logarithm of the minimal angle of resolution; *CDVA* = Corrected distance visual acuity; *CCT* = Central corneal thickness

### Surgical technique

The surgical technique of SMILE procedures was previously described [7, 8]. All surgeries were performed by the same experienced surgeon (XZ) using the Visumax® femtosecond laser system (version 3.1; Carl Zeiss Meditec AG, Jena, Germany). This software was modified for clinical study only and different from the standard version 3.0 software, with spherical correction up to − 14 D and astigmatism correction up to 5 D. Femtosecond scanning was performed with a repetition rate of 500 kHz, pulse energy of 130 nJ, intended cap thickness of 110 or 120 μm. Target refraction in all eyes was plano. The width of the side cut was set to 2 mm at the superior 12 o’clock position. The intended diameter of the lenticule (optical zone) was set to 6.0 to 6.6 mm. The diameter of the cap was set to 1 mm larger than the diameter of the lenticule. All procedures were completed successfully and no intraoperative or postoperative complications were observed. Routine postoperative medications included 0.3% tobramycin, 0.1% fluorometholone, and artificial tears.

### Measurements

Each patient received slit-lamp microscopy, manifest refraction, and tests for uncorrected distance visual acuity (UDVA), CDVA. Wavefront aberrations were measured with the WASCA Analyzer (Carl Zeiss, Meditec, Jena, Germany) under standardized scotopic light settings. Due to the fact that scotopic pupil diameter in several high myopic patients were less than 6 mm, aberrational data were analyzed at 5 mm pupil size using Zernike polynomials. The root mean square (RMS) values of total higher-order aberration (HOA), spherical aberration, coma, and trefoil were calculated. The morphological features of Bowman’s layer were observed using anterior segment spectral-domain optical coherence tomography (OCT) (RTVue, software version 6.2; Optovue, Inc., Fremont, CA). Each measurement consisted of 4 line-scans along the 0°, 45°, 90°, and 135° meridians within the central 4-mm optical zone. Definition and quantitative analysis of the micro-distortions were previously described [9]. In short, the numbers of micro-distortion peaks were counted in each line scan in all 4 meridians. The total number of micro-distortions was calculated by adding all the peaks. All imagings were performed by the same examiner (BQ) and analyzed by another masked investigator (JZ). All measured data were collected preoperatively, 3 months and 6 months postoperatively.***STATISTICAL ANALYSIS***Statistical analyses were performed using Statistical Package for Social Sciences (SPSS) version 19.0 (SPSS, Inc., Chicago, IL). Statistical analysis for visual acuity was based on logMAR units. The t test for two independent samples was used to compare these groups. For repeated measurements, the paired Student t test was used for normally distributed data and the Wilcoxon signed-rank test for abnormal distributed data. The Shapiro-Wilk test was used to test for normality. Age, gender, preoperative central corneal thickness, preoperative mean simulated keratometry, preoperative SE, and lenticule thickness were applied for multivariate linear regression analysis to investigate the possible parameters associated with the total number of micro-distortions. Pearson correlation tests were conducted to investigate the correlation between the total number of micro-distortions and logMAR postoperative visual acuity. *P* value less than 0.05 was considered statistically significant. Sample size calculation algorithms in SPSS was used for sample size calculation. The statistical power of the various values ranged from 0.80 to 0.98 to ensure enough sample size for related analyses. Standardized graphs and terms for refractive surgery results were used in accordance with the recommendations.

## Results

### Safety and efficacy

**Fig. 1:**
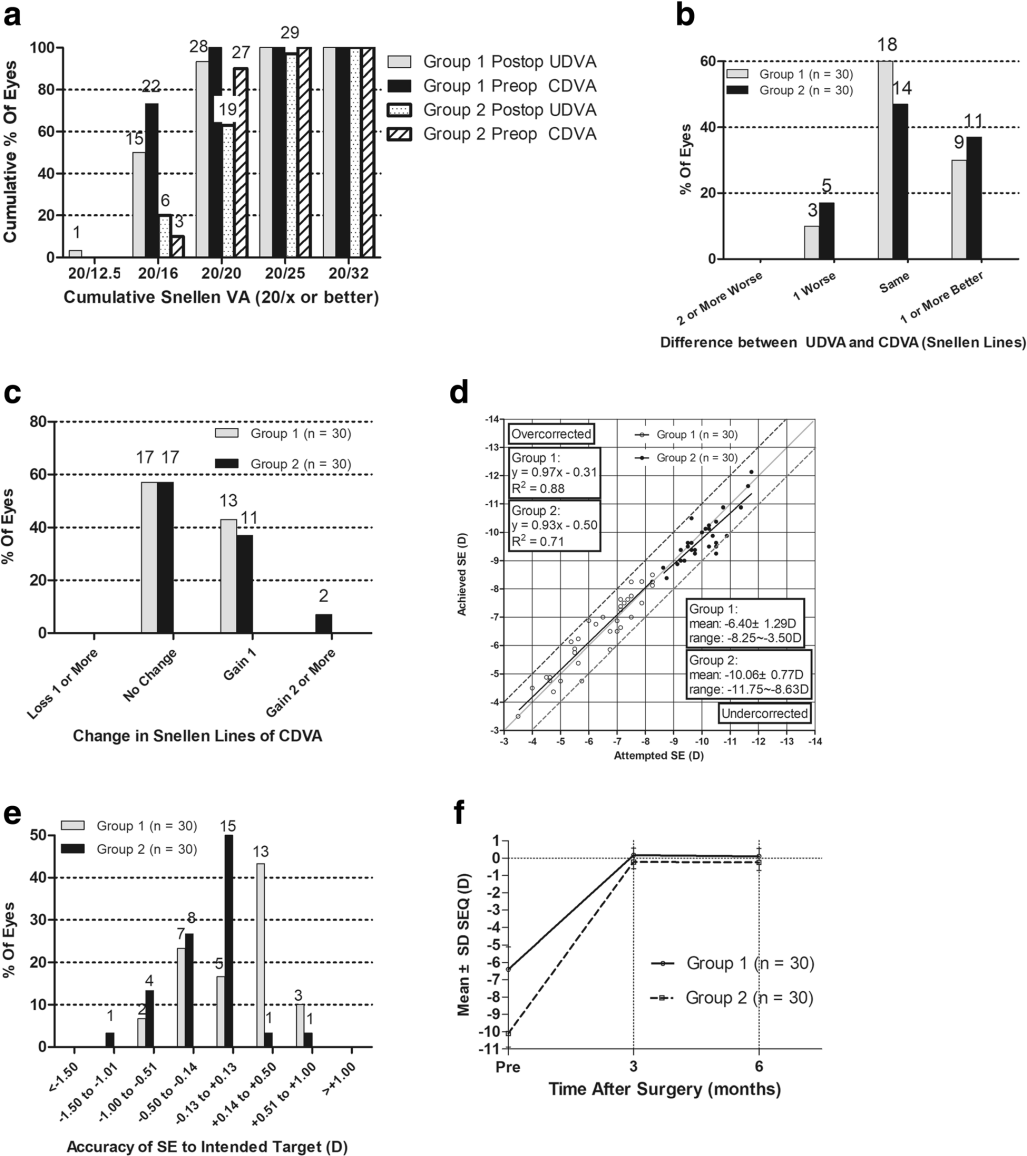
**a** Six-month postoperative cumulative percentage of eyes attaining specified cumulative levels of UDVA comparing group 1 (myopia and myopic astigmatism of − 10 D or less) and group 2 (myopia and myopic astigmatism of over − 10 D) (all eyes had emmetropia as the target refraction). **b** Percentage of eyes comparing UDVA and preoperative CDVA in group 1 and 2. **c** Gain and loss of CDVA in group 1 and 2. **d** Attempted spherical equivalent (SE) refractive change plotted against achieved SE refractive change comparing group 1 and 2. **e** Percentage of eyes attaining specified differences in attempted versus achieved correction comparing group 1 and 2. **f** Postoperative SE refractive change comparing group 1 and 2

All surgeries were completed without intraoperative or postoperative complications. During the 6 months observation, no case of keratectasia was observed. Six months postoperatively, 100 and 97% of eyes (30/30 in group 1, 29/30 in group 2) had postoperative UDVA of 20/25 or better (*P* = 0.20) (Fig. 1a). As shown in Fig. 1b, 26 eyes (87%) in group 1 and 25 eyes (83%) in group 2 had postoperative UDVA either the same as or better than preoperative CDVA. For postoperative CDVA, 17 eyes (57%) showed no change, 13 eyes (43%) gained 1 line in group 1 while 17 eyes (57%) showed no change, 11 eyes (37%) gained 1 line, and 2 eyes (7%) gained 2 lines in group 2 (Fig. 1c). The safety index (ratio between CDVA at 6 months postoperatively and preoperatively) was 1.04 ± 0.10 (range, 0.80 to 1.20) in group 1 and 1.10 ± 0.16 (range, 0.88 to 1.50) in group 2 (*P* = 0.56). The efficacy index (ratio between 6 months postoperative UDVA and preoperative CDVA) was 1.00 ± 0.20 (range, 0.67 to 1.5) in group 1 and 1.03 ± 0.18 (range, 0.60 to 1.2) in group 2 (*P* = 0.071). As shown in Table 2, there was no statistical difference comparing UDVA (*P* = 0.20) and CDVA (*P* = 0.48) between group 1 and group 2 6-month postoperatively. There exists statistically significant difference between these 2 groups in postoperative 6-month spherical equivalent (*P* < 0.01).

**Table 2:** Comparison of postoperative visual acuity and refractions

	6 Months Postoperative		
Parameters	group 1	group 2	*P* Value
LogMAR UDVA			
Mean ± SD	−0.043 ± 0.067	−0.017 ± 0.099	0.20
Range	−0.20 ~ 0.10	− 0.20 ~ 0.20	
LogMAR CDVA			
Mean ± SD	− 0.069 ± 0.047	−0.053 ± 0.073	0.48
Range	−0.10 ~ 0	− 0.20 ~ 0.10	
spherical equivalent (D)			< 0.01
Mean ± SD	0.10 ± 0.46	− 0.25 ± 0.46	
Range	−1.5 ~ 0.5	− 1 ~ 0.88	

*D* = Diopters; group 1 = small-incision lenticule extraction for myopia and myopic astigmatism ≤10 D; group 2 = small-incision lenticule extraction for myopia and myopic astigmatism > − 10 D; LogMAR = logarithm of the minimal angle of resolution; UDVA = uncorrected distance visual acuity; *CDVA* = Corrected distance visual acuity; *SD* = Standard deviation

### Predictability and stability

A scatterplot of the attempted vs the achieved spherical equivalent (SE) correction is shown in Fig. 1d. Six months postoperatively, 83% eyes were within ±0.5 D and 100% were within ±1.0 D of attempted correction in group 1, while 81% eyes were within ±0.5 D and 97% were within ±1.0 D of attempted correction in group 2 (Fig. 1e). There was no statistical difference upon comparing the accuracy of refractive correction at 6-month postoperative between the 2 groups (*P* = 0.45). Figure 1f showed a stable manifest refraction with minor regression during the 6 months period for both groups after SMILE.

### Corneal higher-order aberrations

Upon comparing the postoperative 6-month data between group 1 and group 2, only spherical aberration showed significant difference (*P* < 0.01). Upon comparing the preoperative data with postoperative 6-month data, HOA RMS and coma significantly increased in both groups (*P* < 0.01), and spherical aberration significantly increased in group 2 (*P* < 0.01) but not in group 1 (*P* = 0.44) (Table 3).

**Table 3:** Preoperative vs postoperative HOAs after small-incision lenticule extraction for correcting myopia and myopic astigmatism

	Preoperative			6 Months Postoperative		
Parameters (Mean ± SD)	group 1	group 2	*P* Value	group 1	group 2	*P* Value
HOA RMS	0.23 ± 0.092	0.21 ± 0.067	0.48	0.36 ± 0.11	0.41 ± 0.13	0.09
Spherical Aberration	0.084 ± 0.080	0.071 ± 0.040	0.42	0.094 ± 0.067	0.20 ± 0.12	< 0.01
Coma	0.13 ± 0.091	0.11 ± 0.072	0.33	0.26 ± 0.13	0.25 ± 0.14	0.94
Trefoil	0.12 ± 0.054	0.10 ± 0.067	0.44	0.13 ± 0.073	0.11 ± 0.070	0.34
	Preoperative compared with 6 Months Postoperative					
Parameters	group 1		group 2			
(Mean ± SD)	Mean Difference	P Value	Mean Difference	P Value		
HOA RMS	0.13 ± 0.11	< 0.01	0.19 ± 0.15	< 0.01		
Spherical Aberration	0.010 ± 0.078	0.44	0.12 ± 0.14	< 0.01		
Coma	0.13 ± 0.13	< 0.01	0.14 ± 0.14	< 0.01		
Trefoil	0.015 ± 0.071	0.22	0.01 ± 0.081	0.74		

*SD* = Standard deviation; *D* = Diopters; group 1 = small-incision lenticule extraction for myopia and myopic astigmatism ≤10 D; group 2 = small-incision lenticule extraction for myopia and myopic astigmatism > − 10 D; HOA RMS = root mean square of total higher-order aberration

### Micro-distortions in BOWMAN’S layer

**Fig. 2:**
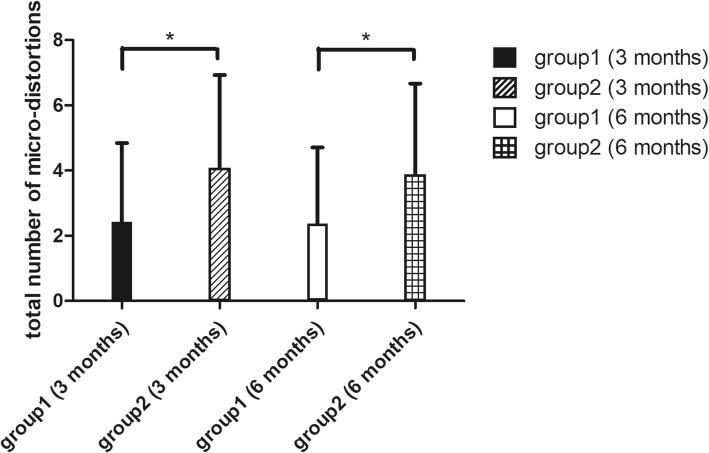
Comparison of total number of micro-distortions in Bowman’s layer in group 1 (myopia and myopic astigmatism of − 10 D or less) and group 2 (myopia and myopic astigmatism of over − 10 D) 3 months and 6 months after small incision lenticule extraction (SMILE). * = statistically significant

In group 1, micro-distortion was observed in 60% eyes 3 months postoperatively and 53% at 6 months postoperatively. In group 2, micro-distortion was observed in 80% eyes 3 months postoperatively and 77% 6 months postoperatively. As shown in Fig. 2, the total number of micro-distortions was 2.43 ± 2.42 in group 1 3 months postoperatively, which was significantly less than that of 4.07 ± 2.86 in group 2 (*P* = 0.02). More micro-distortions were observed in group 2 (3.40 ± 2.66) than in group 1 (2.07 ± 2.29) (*P* = 0.041) 6 months postoperatively. There does not exist statistically significant differences between the total number of micro-distortions 3 and 6 months postoperatively between group 1 (*P* = 0.07) and group 2 (*P* = 0.25).

Multivariate linear regression analysis showed that the total number of micro-distortions in SMILE operated eyes was associated with preoperative SE (b = − 0.11, *P* < 0.01) and lenticule thickness (b = − 0.05, *P* < 0.01). The total number of micro-distortions was not correlated with either 6-month postoperative UDVA (*P* = 0.14) or CDVA (*P* = 0.77).

## Discussion

It is widely accepted that the standard small-incision lenticule extraction procedure is capable of treating eyes with spherical error of no more than − 10 D, and that SMILE has shown promising results in terms of visual outcomes, safety, efficacy and predictability [4, 10–12]. The modified Visumax® femtosecond laser system in this study was capable of treating spherical error up to − 14 D. In our study, we presented preliminary results comparing eyes with myopia and myopic astigmatism of ≤ − 10 D and > − 10 D undergoing SMILE procedure.

In terms of safety, there was no statistical difference upon comparing the safety indices between two groups. Previous studies reported the safety indices of SMILE up to − 10 D, being 1.01 ± 0.05 [13] and 1.09 [14] at 3 and 1 month postoperatively, which was similar to our result of 1.04 ± 0.10 in group 1 and 1.10 ± 0.16 in group 2. Several studies reported that the low to moderate myopic eyes treated with SMILE had the rate of CDVA loss for one or more lines of 1.1% [1] and 1.67% [13]. In our study, no eyes lost l or more lines of CDVA. For efficacy comparison, previous results regarding SMILE up to − 10 D reported the efficacy indices of 1.04 ± 0.20 [13] and 1.09 [14] at postoperative 3 and 1 month respectively. Our results of 1.00 ± 0.20 in group 1 and 1.03 ± 0.18 in group 2 were consistent with these results, also showing no statistical difference between the two groups. For previous postoperative results of SMILE up to − 10 D, 62% had an UDVA of 20/20 or better, and 93% of 20/25 or better [15–18]. In our study, 94% (group1) and 63% (group2) eyes had an UDVA of 20/20 or better, and 100% (group1) and 97% (group2) had an UDVA of 20/25 or better. As for predictability, studies of SMILE in treating preoperative SE of ≥6.00D myopic eyes reported a predictability of 80% [19] and 88% [20] within ±0.50D of target refraction, and of 94% [19] and 97% [20] within ±1.0D, respectively. These results were consistent with ours of 83% (group 1), 80% (group 2) within ±0.50D, and 100% (group 1), 97% (group 2) within ±1.0D (Fig. 1e). Our results showed no statistical difference of UDVA and CDVA between group 1 and group 2. All these results suggested that SMILE in correcting myopia and myopic astigmatism of > − 10 D had similar results compared with those ≤ − 10 D in terms of safety, efficacy, and predictability. For corneal wavefront aberration analyses, HOA RMS and coma increased in both groups, but spherical aberration only increased in group 2 and trefoil did not increase in either group preoperatively and 6 months postoperatively. Previous literatures showed conflicting results regarding changes in HOA before and after the surgery. Some studies indicated that HOA RMS, spherical aberrations, and coma increased postoperatively [20, 21]. Others found that only HOA RMS and coma increased, while spherical aberration remained stable [7, 9, 22]. There might exist several reasons for such discrepancies. First, different instruments for measurements and different scotopic environments could affect the results. Second, aberrational data were evaluated under different pupil sizes. Using a smaller pupil size for analysis might lose some information. But in some extreme myopic eyes, scotopic pupil size could be smaller than the intended pupil size for analysis, hence using a smaller analytical pupil size was inevitable. In this study, we found spherical aberration being higher in group 2 compared with group1 6-month postoperatively. This might be due to the fact that a smaller designated optical zone was applied for higher myopic correction. Moreover, a thicker stromal lenticule needed to be excised, which caused a larger change in anterior corneal asphericity and induced more spherical aberration. Previous PRK and LASIK studies also indicated that the optical zone had a significant impact in spherical aberrations [23–26]. Further studies should be conducted to explain the spherical aberration increase for high level myopic correction, as well as its potential influence on visual quality such as contrast sensitivity and intraocular scattering. For coma increase, the SMILE platform currently lacks a tracking system. Minor decentration was inevitable, which might have minimal effect in postoperative visual acuity but could potentially induce coma.

**Fig. 3:**
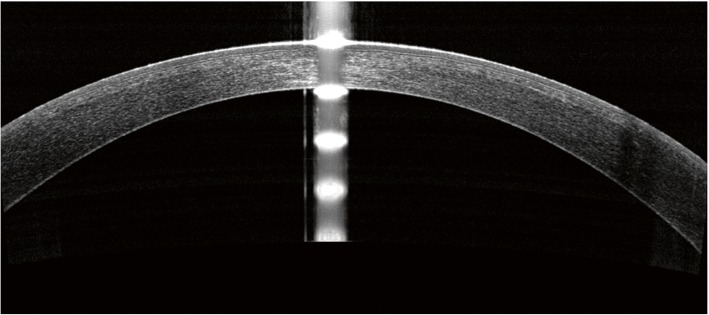
The optical coherence tomography image of cornea 6 months after small incision lenticule extraction (SMILE) showing rugged Bowman’s layer with 1 micro-distortion marked with arrowhead and the epithelium with uneven thickness but a smooth corneal surface

In previous literature, the longest follow-up time for Bowman’s layer micro-distortions observation after SMILE surgery was 3 months, which showed that the total number of micro-distortions decreased overtime [27]. In this present study, we further extended the observation time to 6 months postoperatively, finding that micro-distortions still existed but the total number remained stable for 3 and 6 months postoperatively. This suggested that the corneal shape became stable after certain time postoperatively, and the micro-distortions could still remain. More micro-distortions were found in group 2, and the total number of micro-distortions was associated with preoperative SE and refractive lenticule thickness. These findings suggested that patients with higher myopic correction tend to have more micro-distortions. The results further confirmed certain previous findings [8, 9], which bring out the hypothesis that micro-distortions might be due to mechanical changes in cornea and greater difference in curvatures between stromal bed and corneal cap after the extraction of thicker lenticule. The total number of micro-distortions were not correlated with UDVA or CDVA. This is one of the evidence adding to the efficacy, safety and the fast visual recovery of the SMILE procedure [2, 3, 28, 29]. On the OCT images, although Bowman’s layer became rugged with micro-distortions after SMILE, the epithelium had uneven thickness but a smooth surface to ensure good refractive quality of the whole cornea (Fig. 3). Although the smooth corneal surface could explain the good visual acuity after SMILE surgery, further investigations should be conducted to reveal the potential impacts of micro-distortions in visual quality.

Our study has several limitations. We presented a comparatively small sample and the follow-up time is short. We did not analyze the vertical and horizontal coma separately when doing HOA coma analysis. Also, we did not compare the procedures with other types of surgeries such as LASIK. We are currently conducting a further study with larger sample and different types of surgeries comparisons.

## Conclusions

In conclusion, our results demonstrated that SMILE for the treatment of myopia and myopic astigmatism of > − 10 D were good in visual outcomes in terms of safety, efficacy, predictability, and stability while having slightly under-correction compared to those ≤ − 10 D. Higher myopic treatment tends to induce more spherical aberrations. No vision-threatening complications occurred throughout the 6-month follow-up period. Micro-distortions in Bowman’s layer were associated with preoperative SE and lenticule thickness, but had no impact on visual and refractive outcomes. This procedure seems promising in terms of extending the indications of SMILE.

## Data Availability

Available upon request from the first author; Dr. Bing Qin.

## Abbreviations

CDVA: Corrected distance visual acuity
D: Diopters
HOAs: Total higher-order aberrations
LASIK: Laser in situ keratomileusis
OCT: Optical coherence tomography
RMS: Root mean square
SE: Spherical equivalent
SMILE: Small-incision lenticule extraction
UDVA: Uncorrected distance visual acuity
